# The Rise of the Guest Editor—Discontinuities of Editorship in Scholarly Publishing

**DOI:** 10.3389/frma.2021.748171

**Published:** 2022-01-18

**Authors:** Marcel Knöchelmann, Felicitas Hesselmann, Martin Reinhart, Cornelia Schendzielorz

**Affiliations:** ^1^German Center for Higher Education Research and Science Studies, Research System and Science Dynamics, Berlin, Germany; ^2^Department of Information Studies, University College London, London, United Kingdom; ^3^Robert K Merton Centre for Science Studies, Humboldt-Universität zu Berlin, Berlin, Germany

**Keywords:** scholarly publishing, editorship, knowledge production, scholarly communities, publishing platforms, guest editor, open access

## Abstract

Scholarly publishing lives on traditioned terminology that gives meaning to subjects such as authors, inhouse editors and external guest editors, artifacts such as articles, journals, special issues, and collected editions, or practices of acquisition, selection, and review. These subjects, artifacts, and practices ground the constitution of scholarly discourse. And yet, the meaning ascribed to each of these terms shifts, blurs, or is disguised as publishing culture shifts, which becomes manifest in new digital publishing technology, new forms of publishing management, and new forms of scholarly knowledge production. As a result, we may come to over- or underestimate changes in scholarly communication based on traditioned but shifting terminology. In this article, we discuss instances of scholarly publishing whose meaning shifted. We showcase the cultural shift that becomes manifest in the new, prolific guest editor. Though the term suggests an established subject, this editorial role crystallizes a new cultural setting of loosened discourse communities and temporal structures, a blurring of publishing genres and, ultimately, the foundations of academic knowledge production.

## Introduction

It is a truism to claim that scholarly publishing is undergoing profound changes. Visible transformations are driven by a diversity of developments such as the increasing impact of digital technology, new modes of commercialization, changing publishing practices such as preprinting or open access, and new subject formations concerning authorship, reviewership, or, indeed, editorship. While these developments are heterogeneous, they are at the same time also interconnected in many ways. Their interplay becomes visible in specific phenomena, such as the emergence and rise of so-called mega-journals (e.g. Siler et al., 2020), which involves changes in terms of publishing organizations (Wakeling et al., 2017a,b), authorship practices (Wakeling et al., 2019), peer review (Björk and Catani, 2016), and others. Such phenomena are also visible in terms of the structuring or regularity of publishing as, for instance, the “number of special issues was over twice the number of ordinary issues in 92.45% of the *MDPI*-journals” assessed during an analysis (Oviedo-García, 2021a,b, p. 410).^1^ On average, the *explosion* of special issues visible at *MDPI* ridicules the meaning of the word *special* since it grows on the exponential increase of that which is supposed to be special within the ordinary. The “army of *MDPI*-invited Guest Editors” (Crosetto, 2021) is the necessary condition for this rise to work, or be manageable cost-effectively. A reflexive result of such an organizational publishing policy is the shifting of publishing incentives and reputation gain, which unprecedentedly emphasized the role of a prolific guest editor.

In this contribution, we take a look at some of the ways these developments connect to and interact with one another by focusing on the role of the guest editor as one such cross-cutting phenomenon. This focus on the guest editor provides a unique perspective that has previously been quite neglected in research. The figure of the guest editor here serves as a conceptual lens through which some of the transformations within publishing practices come into view. We identify a contemporary subject formation of the guest editor as being both a symptom and a driving force that brings together some of these seemingly disparate developments.

Discussing these transformations through the guest editor, therefore, provides us with a burning glass which lays bare some of the mechanisms that stay invisible at the level of phenomena themselves. The figurative *rise* is a *crystallization* of cultural settings in this sense. Moreover, we do not claim to excavate a truth of historical developments. Much rather, we treat guest editorship and traditional editorship as two conceptual polar opposites, with concrete examples of publishing practices most likely falling somewhere in between. We use our focus on this crystallization to generate a number of hypotheses about current and future developments of scholarly publishing so that it may trigger further research in this direction. This perspective article may serve as a starting point for further research and debate. We proceed as follows: firstly, we highlight characteristics of a traditional mode of editorship. Secondly, we contrast this traditionalist subject with the contemporary guest editor. Thirdly, we connect these new characteristics to wider developments of publishing platforms.

## Curation and Community: The Traditional Mode of Editorship

Editorship in scholarly publishing means to make texts appear and thus to place content as well as authors in a context. Editorship is not primarily a work on text, but on context. The fact that a collection of texts is edited usually implies that this compilation was undertaken consciously, thoughtfully, and purposefully; i.e. it is not arbitrary. Therefore, editorship is usually accompanied by the claim that assembled textual contents can be related to one another in some meaningful, structured, and comprehensible way. It involves curatorial practices that require systematization, selection, and bundling (Lindsey, 1976; Bell and Bridgman, 2019). Consequently, editorship goes hand in hand with the identification of topics, the delimitation of subject areas, the structured preparation of commonalities and differences in research approaches, as well as with the presentation of homogeneous or heterogeneous positioning in discourse. Three aspects stand out when the role of the editor is to be defined:

Firstly, the journal's name and published content and the names of the editors who select the content to be published are mutually dependent. This extends historically as well as functionally, for instance, to the recruitment of subsequent editors. Social validity and networks are key factors in editorial board member recruitment (Miniaci and Pezzoni, 2020). Editorial boards tie names from a field to a journal and at the same time the name of the journal is linked to the positions of the editors in the field. The gatekeeping role of the editor thus is not limited to a selection of content, but also includes a selection of people who can associate themselves with a given journal, a fact that is often critically highlighted with regard to unequal representation within academia (e.g. Goyanes, 2019; Albuquerque et al., 2020). The founding of a journal is a special case for a limited period of time, in which the overlap between journal profile and editor/founder profiles is particularly large.

Secondly, based on the intersection of the profiles of journal and editors, the journal's brand is shaped by its editorship. This applies even more insofar as the contouring and topic setting of the journal by means of its editors also appeals to a group of scholars, that is the community. This community serves equally as an audience in the sense of readership, which constitutes the potential pool of reviewers to recruit as well as future authors to attract. Editorship can, thus, establish a mode of visibility, which contributes to the forming, strengthening, or establishment of a community (See examples such as Science & Technology Studies, or Valuation Studies: Kjellberg and Mallard, 2013). Consequently, the connection of journals to a community of scholars is a defining feature of scientific disciplines (Stichweh, 1992).

Thirdly, editorship impacts the generation, accumulation, and diffusion of reputation by making visible and profiling a topic as well as an associated community (Lange and Frensch, 1999; Fyfe and Gielas, 2020). In this respect, editorship can also serve as a sort of reputation guarantor. This encompasses a symbolic guarantee for the relevance and quality of both the journal's claim and the articles published in it. This function of editorship can also be conceived of as in the sense of a mediated consecration—mediated, since the functioning of consecration is usually borne by and supplemented with the informed opinions of (peer) reviewers. Reviewing serves as a key influence on editorial practices. It neither discharges the editor as decision-making authority nor does it supersede it.

## Loose Clusters and Temporal Structures: The Rise of The New Guest Editor

In contrast to the traditional model of editorship outlined above, the prolific, new guest editor takes on editorial responsibilities on a short-term basis, usually for a single journal (special) issue. This typically has a relatively narrow thematic focus. From the journal's perspective, calling on guest editors rather than professional editorial staff for fulfilling crucial editorial duties represents a form of outsourcing. While more managerial or organizational tasks such as rights management or production remain with the journal's or publisher's staff, the pivotal scholarly tasks of curation and community building are fulfilled by the guest editor.

With regard to the curation of scholarly content, the reliance on guest editors goes hand in hand with an opening of the thematic profile of the traditional journal. Here, a journal's profile unfolds as a temporal sequence of thematic foci or topics that are relatively narrow but not necessarily strongly related to one another. This comes with distinct effects on scholarly discourse. Rather than being characterized by steady, (more or less) consistent thematical and disciplinary developments, scholarly discourse becomes structured by event-driven, single-occurrence clusters that appear and dissolve across disciplinary boundaries. Novelty, timeliness, or relation to current events become essential criteria for the success (or failure) of such discourse clusters. This also comes with an increased reliance on the guest editor's ability to recognize or create timely successful topics, as they can no longer draw on an established discourse to steadily generate content, or attract authors and readers. Topics might also look decidedly different if they are specifically created around current events that cut across disciplinary boundaries, instead of being generated by ongoing disciplinary debates.

This transformation is mirrored on the level of the respective scholarly communities, which experience a process of dissolution as well. Journals are no longer connected to a relatively stable group of authors, reviewers, and readers that form a particular community. Instead, those groups become event-driven and somewhat nomadic, much like the topics they concern themselves with. They temporarily gather around a particular question that a particular guest editor addresses in a particular issue, and subsequently dissolve, or move on to other journals and publishing venues for other special issues. This dissolution obviously is not solely the result of a rising importance of special issues, but triggered by a number of factors. An early factor is the shift from (primarily) print to (primarily) online journals which allow for article-level access instead of issue-level access, and which also led to a push for article-level metrics (Fenner, 2013). As a consequence, the ability of guest editors to address or generate these temporary audiences becomes essential for the journal, the topic, as well as the audiences themselves. While community building is a crucial responsibility for the traditional editor, too, this process is cyclical rather than continuous for the guest editors, and it takes place under conditions of strongly increased time pressure. The groups constituting around topic clusters of special issues—even if they constantly move from one journal to the next and so on—are thematically more narrowly defined than the relatively broad disciplinary communities that often form a traditional journal's audience.

With the rise of the guest editor, the generation, accumulation, and distribution of academic reputation also becomes much more precarious for journals. Not being able to draw on a stable editorship as a reputation guarantor, journals either must rely heavily on the prestige and reputation generation abilities of a host of changing guest editors, or themselves become the brand^2^ that generates reputation. Becoming their own reputation generators is only possible for a small number of high impact-journals (or their branded journal families), such as *Science* and *Nature*. Their reputation is mostly independent of specific topics and spans a wide range of different communities. In this case, guest editors profit more from being associated with a journal than vice versa. For most journals, however, establishing themselves as brands becomes more difficult, which may lead to journals moving more and more into the background and taking on a role closer to a sole publishing platform, rather than a brand defined by curation and community.

Historically, we would expect the guest editor to operate in this “middle ground,” where the theme of a special issue fits well with the thematic profile of a journal and where the reputation of the guest editor ties the thematic community to that journal. Outside of that middle ground, the role of guest editors seems to make less sense. High ranking journals and guest editors seem to be an odd couple, at least in the long run, as the thematic clustering of articles will be detrimental to the goal of only publishing the best work within a research field and, consequently, reject most submissions. Curating a special issue in such a context will be difficult, as thematic considerations regularly conflict with aspirations for quality and gatekeeping. Conversely, guest editorship in very low-impact journals may appear equally odd. In a context of high acceptance rates, the work of curating a special issue may have little reputational benefit for the thematic community. It does so only for scientists or scholars who strive to gain from being made visible at any price, irrespective of a journal's or publisher's low reputation, and who cannot serve at more visible or esteemed venues. The reputational gain of such an editorship may be questionable beyond the mere formality of authorship since the low-impact journal is by definition rather not visible, and so are its authors and editors. According to such a view that aligns well with a traditional publishing system where there is a clear reputational hierarchy of scientific journals, we would expect guest editorship to be frequent “in the middle.” However, end-points of this spectrum—high and low ranking journals—have seen a considerable shift toward platform models of publishing. On the high-ranking side, brands like *Nature* have become a whole system of interconnected journals, both broad and thematically specific, while on the low-ranking side, platforms provide publishing solutions with low rejection rates and servicing multiple disciplines at once. These modes of platforms meet where they both offer under-differentiated, so called mega-journals. While not really “in the middle,” these platform contexts open up a space where guest editorship is performed in new ways.

## Publishing Platforms, Publishers, and the Role of the Guest Editor

The shift in journal publishing practices is characterized by a new mode of activity, or activisation. This development of activisation is marked by earlier practices of editorial work being side-lined by practices of acquisition. Consider how journals were best described by a sort of passive activity in the past (being active primarily indirectly through editorial facilitation). Special issues and guest editors enable journals to be active as direct players. They engage in agenda setting as they propose topics and actively acquire authors for these as well as for regular paths of submission of their journals.

The twist, however, is constituted by the fact that this direct activity—the acquisition as well as the agenda setting—often stems from guests, and is therefore constituted without the inhouse editorial team, or only so mediated by them. It is in this respect important to note that the rise of the guest editor and associated developments of outsourcing have a strong impact on the mode journals operate in, in the sense described here.

This development even transcends the agenda setting within the subject area of a journal and affects scholarly fields in that journals themselves are launched. Publishers that provide a publishing platform (such as *MDPI* or *Frontiers*) can easily expand their portfolio of journals and, thus, publishing brands by seeking potential new sub-fields, combinations of existing fields, or new geographic (i.e. internationalizing or localizing) areas of a field. By so doing, they provide a new publishing site for this—pre-emptively moderated or constituted—community. As this sub-field may not yet exist, there is, furthermore, not yet a corresponding discourse community that negotiates relevant epistemes of that sub-field.

By launching the journal, the publisher substantially contributes to laying the grounds for the existence of that sub-field by means of a potential communicative site of it. Of course, the launching of a new journal does not necessarily constitute a new sub-field. Such development always needs to correspond to a potential in the subject area: a potential claiming that the existence of the sub-field is epistemically possible and desirable by a critical number of scholars. It is, therefore, the role of early editors that shape the role the new journal may take on and whether the journal can fulfill such criteria. Accompanying the early years of journals with special issues seems advantageous for the publisher since ‘the publication of special issues appears to offer the potential benefits of attracting submissions, increasing profits, and improving impact' (Repiso et al., 2021, p. 599). By such means, the guest editor is accorded a foundational role for laying grounds for a journal's agenda and its potential success.

For publishers, such launches are primary paths of business development more generally. Indeed, for such platforms as *MDPI*, “the fact that the number of special issues in JCR-indexed *MDPI*-journals is so much higher than the number of ordinary issues per year coupled with their constant increase since 2018 inevitably awakens suspicions of a lucrative business aim” (Oviedo-García, 2021a,b, p. 9). These provide decreasing marginal costs: each journal on a publishing platform makes use of the same, once-developed technological means without further required production investment. This is comparable to the printing press for which, once it is set up, it makes sense to keep the press running in order to reach maximum capacity and decreasing marginal costs. As a bulk of the cost of publishing stems from expenditure for human resources (editors, editorial managers and assistants, web developers, etc.), outsourcing (some of) the editorial work to guest editors further supports these cost-cutting measures, at least up to a degree. As further costs regarding editorial work are marginalized and the cost-intensive practices of field correspondence and acquisitions are outsourced, and considering that multi-journal holding publishers are run on an open access model, the new journal provides income that is lucrative in terms of returns on investment. To be sure, the effect of such cost-saving is dependent on the scalability of the practice. Two issues are important to note in this respect.

Firstly, brands perform as distinctive indicators. They can be compared to keywords on a platform on which, without such keywords, the platform would be merely a single mega-journal. This is an inversion of traditional developments again. Traditionally, there have been distinct, physical journals comprising of their own style in terms of layout, editorial work, or graphical nuances, and depending on distinct editorial work that relates to specific audiences and writers. Such journals had to be incorporated into a digital platform posteriorly, a development that usually had to cut out certain stylistic elements as well as combined editorial works within editorial offices. The digital was, then, an accessory in the social and humanistic, and the predominant mode of existence in the scientific disciplines. Nevertheless, it had run as a distinct brand that had been incorporated into the digital platform.

The newly launched journals within multi-journal publishing platforms are born as efficient means within this platform. Most journals here are similar if not equal to all others on the same platform in terms of layout, style, or submission systems. The distinctive feature (apart from the content) is the brand, in terms of both its graphics as well as name, and its function for a discourse community. It is, in sober terms, more or less a keyword with a brand identity among many others on the platform. Users can choose a keyword to reach areas of content—i.e. choose a journal. The brand identity may appear to be more complex as it involves graphics, descriptions, editorial responsibilities, etc. But these are merely internal complexities for a journal. In terms of the publishing platform, these complexities are dissolved as the journal brand and all its residual constitutive features come down to being a differentiator on a platform.

Secondly, this also contributes to the issue that publishing genres blur or their distinctions dissolve. Especially along the lines of special issue and edited collection (edited volume or the German *Sammelband*), the genres journal and book seem to converge. A journal's special issue is characterized by its speciality in several respects. It may step out of the periodicity of the journal and suspends, or side-lines, the usual submission guidelines and content focus in that a particular subject focus is being picked which internally unites all articles to be published. An edited collection approaches these very characteristics from a somewhat oppositional perspective: the book usually is non-periodical by nature and, except for specific series, books in a programme are not connected by a mutual focus as do articles in a special issue. They are standalone publications, even though series may bind them because of a topical nearness. Journal articles are never standalone publications.

And yet, if a journal repeatedly publishes special issues, how different is it from a book series that publishes collected editions in a digital environment? Both special issue and collected edition share their essence of being a topically united collection of shorter pieces that appears as a seemingly standalone publication, both are accessible on larger publishing platforms by a diversity of means (title, search functions, topic references). They are to be downloaded by article or by compressed PDF, both commissioned by scholars from within the field, the publisher merely providing technological means and brands in response to processing fees. This becomes ever more visible in the form of article collections, also termed overlay issues, with which publishers actively collate articles post publication. This can even be seen as a grounded special issue as publishers—whether editors, guest-editors, or author collectives—may take on a curating role as the articles published may suggest. It is in this respect to ask: what is the specificity of these publishing genres other than their historical constitution?

The role of the guest within editorial praxis is tangible throughout the aspects accounted for here. These aspects are not all bound to a causal connection in that the guest editor is posited to be the reason for the developments described. But more often than not, the guest editor is at least a crucial cog in the scholarly publishing machine—seemingly minor but with tremendous impact on the development. Without the guest editor, publishing platforms would not be able to thrive the way they do since their cost-cutting measures have cut out the essential links and expertise the guest editor contributes today.

Publishers would not be able to stay connected to scholarly fields and their developments, both in ways to connect to intellectual resources such that journals become, or stay, relevant as essential communicative sites, to lure in submissions and guide them to relevant audiences; and in ways to stay alert to relevant meta-developments, the new sub-fields and *topoi* to explore and to, potentially, serve and exploit. It is the guest editor—the scholar-seeking-network-and-reputational-esteem—who provides both these means. And yet, whether the enlargement of social and symbolic capital are fulfilled by such a role is a different question, one that can be answered only with an eye on the failure or success of the publishing platform itself, indicating again the circular enforcement of publishing.

## Conclusion

On the surface, editors and guest editors may appear to remain established subjects, bounded, as they were, by well-known scholarly communication practices. At closer inspection, however, we can decipher a shift of practices which results in a shift of meaning. It is this shift of meaning that we have accounted for in this article. While, to be sure, there never were unequivocal definitions of what it means to be an editor, the shift of meaning that we describe goes beyond variations of the past. The rise of the new, prolific guest editor showcases a crystallization of new cultural and structural settings that scholars, scientists, and staff concerned with scholarly publishing in a wider sense need to be aware of.

This awareness is required as the new cultural settings fundamentally affect how academic knowledge is being produced. It touches on questions that have to concern all involved in contributing to knowledge production, such as: What are reasons to publish? What function does a scholarly publication have? It is a shift that exemplifies altered foundations of academic knowledge production. And it connects to several cross-cutting themes that may guide further research. We see at least four strands where empirical analysis would allow for a better understanding of these transformations.

Firstly, the development from thorough curation to lose clustering needs to be studied empirically in order to determine to which extent this change entails an uncoupling of production and reception of scholarly discourse with regard to the contextualization and interrelation of contributions to a special issue or edited book. In this vein, the guest editor might even be somewhat of a transitory figure, with journals and publishers only retroactively clustering articles into “collections” after publication, thus cutting out the guest editor's responsibility for pro-actively generating content with a specific thematic profile.

Secondly, the consequences and repercussions of these transformations in the relation of publishers, publishing platforms, and (guest-)editors for reputation and the accumulation of social and symbolic capitals urgently call for further examination (see also: Desrochers et al., 2018, p. 239–240). Which practices, performances, processes, interrelations, and exchanges generate reputation for whom? How is it diffused? Where does reputation arise and condense to such an extent that it serves for distinction and can be powerfully asserted and multiplied? To whom—individuals, organizational actors such as journals or publishing platforms, or research and higher education institutions such as universities, research hubs—is distinctive reputation being attached and ascribed?

Thirdly, we need to explore what this change in editorial work and curation means for the reception and impact of scholarly knowledge production in the academic community as well as in society. Consider that the function of curation in journals is also to calibrate the reading time of the discipline or community addressed (Hirschauer, 2004, p. 72). By selecting articles for publication in high-impact journals, the claim of validity for scholarly knowledge is increased, since it was examined and deemed relevant by authorities in the field in the course of peer review and the editor's comments. The bundling of contributions in edited volumes also fulfilled a thematically narrow orientation for a community. Given the growth of science and scholarship, the need for such sorting and orientation guidance has by no means diminished. Consequently, the question arises how the dissemination and dispersion of trusted knowledge will be affected if this pre-structuring and curation of the information stock on the level of journal volumes or anthologies risks disappearing. Does the circulation of trusted knowledge become more open, for example, to cross-disciplinary reception and expand the possible scope for original linkages, e.g., the subjectively perceived freedom and recognition of inter- and transdisciplinary collaborations, relationships, and networks? Does it become more random and arbitrary, and does a disciplinary and thematically less pre-structured reading bear the risk of quality losses, e.g. blind spots, insufficient consistency and coherence of the perceived body of knowledge, or lack of overview of the state of research relevant to the topic? And last but not least, what alternative guidance is at hand? Is a return to an orientation toward authors or groups of authors as central kernels of debates to be expected? Such a drift toward an author-centered reception could provide some explanation for the super-visibility (Brighenti, 2007, p. 330) that some researchers achieve supported by the expanded channels of knowledge communication.

Fourthly, the fluidity of editorship made visible here may trigger changes that may allow for more diverse settings of power. Traditional editors have power by means of their curation, a point that is often criticized as hampering social and epistemic diversity in publishing (e.g. Collyer, 2018). The diversity of editorial boards, likewise, is disputed (Goyanes, 2019; Albuquerque et al., 2020). Here, guest editors might bring some sorely needed equity into global scholarly publishing. Questions of power are also pertinent when considering the relationship between traditional editors and guest editors: here, traditional editors might very well still be involved even when a special issue is curated by a guest editor, and hence retain some of their decision-making power as well as their influence over the thematic focus of a journal. Future research may explore whether the rise of the guest editor indeed leads to a shift of power or whether it only strengthens the traditional subject in a cultural backlash. This aligns well with the discussions of community-owned infrastructures (Adema and Moore, 2018; Barnes and Gatti, 2019; Moore and Adema, 2020) within the larger debates about open access. Notions of democratization are common in such discourses, even though larger developments rather create a democratic myth as power structures remain as they are (Knöchelmann, 2021).

In any case, these transformations hold opportunities with regard to the opening of new spaces of possibility and the cross-disciplinary unfolding of scientific knowledge production and reception from narrowly defined, pre-traced paths. Further research will also need to bear in mind and address the risks concerning research quality, scientific standards, instrumentalization and premature solutionism, which these transformations also entail.

## Data Availability Statement

The original contributions presented in the study are included in the article, further inquiries can be directed to the corresponding author.

## Author Contributions

All authors listed have made a substantial, direct, and intellectual contribution to the work and approved it for publication.

## Funding

This project was funded by German Ministry for Education and Research (BMBF), grant no. 01PW18015.

## Conflict of Interest

The authors declare that the research was conducted in the absence of any commercial or financial relationships that could be construed as a potential conflict of interest.

## Publisher's Note

All claims expressed in this article are solely those of the authors and do not necessarily represent those of their affiliated organizations, or those of the publisher, the editors and the reviewers. Any product that may be evaluated in this article, or claim that may be made by its manufacturer, is not guaranteed or endorsed by the publisher.

## Footnotes

^1^Please note that, at the time of writing, this article is subject to an Expression of Concern: Oviedo-García (2021a).

^2^A brand may refer to journal, publisher, or (book) series—always depending on how it is perceived by an audience such as a discourse community. In this respect, brands can leverage each other as is visible with well-renowned journal families such as *Nature*, or with journals or monograph series that are well-regarded themselves within also esteemed publishers such as some university presses. Likewise, one brand may also harm the other such that a journal's esteem among an audience may fall since it is published with a corporate publisher [see, for instance, the debate on *Elsevier*: Heyman et al. (2016)]. Thus, it cannot be clearly disentangled what specifically is being referred to as a brand.
